# A serum protein signature at the time of Uveal Melanoma diagnosis predicts long-term patient survival

**DOI:** 10.1186/s12885-023-10757-x

**Published:** 2023-03-27

**Authors:** Christina Herrspiegel, Flavia Plastino, Emma Lardner, Stefan Seregard, Pete A. Williams, Helder André, Gustav Stålhammar

**Affiliations:** 1 St. Erik Eye Hospital, Eugeniavägen 12, 4078, Stockholm, 171 64 Sweden; 2 Department of Clinical Neuroscience, Division of Eye and Vision, Karolinska Institutet, Tomtebodavägen 18A, Stockholm, 171 77 Sweden

**Keywords:** Uveal melanoma, Choroidal melanoma, Liquid biopsy, Serum sample, Proteome, Prognosis, Survival, Metastasis, serUM-Px

## Abstract

**Purpose:**

To develop a prognostic test based on a single blood sample obtained at the time of uveal melanoma diagnosis.

**Methods:**

83 patients diagnosed with posterior uveal melanoma between 1996 and 2000 were included. Peripheral serum samples were obtained at diagnosis and kept at -80 °C until this analysis. Protein profiling of 84 cancer-related proteins was used to screen for potential biomarkers and a prognostic test that stratifies patients into metastatic risk categories was developed (serUM-Px) in a training cohort and then tested in a validation cohort.

**Results:**

Low serum leptin levels and high osteopontin levels were found to identify patients with poor prognosis and were therefore selected for inclusion in the final test. In the validation cohort, patient sex and American Joint Committee on Cancer stages were similarly distributed between the low, intermediate, and high metastatic risk categories. With increasing metastatic risk category, patients had shorter metastasis-free- and overall survival, as well as greater cumulative incidence of uveal melanoma-related mortality in competing risk analysis (*P* = 0.007, 0.018 and 0.029, respectively). In multivariate Cox regression, serUM-Px was an independent predictor of metastasis with tumor size and patient sex as covariates (hazard ratio 3.2, 95% CI 1.5–6.9).

**Conclusions:**

A prognostic test based on a single peripheral venous blood sample at the time of uveal melanoma diagnosis stratifies patients into low, intermediate, and high metastatic risk categories. Prospective validation will facilitate its clinical utility.

**Supplementary Information:**

The online version contains supplementary material available at 10.1186/s12885-023-10757-x.

## Introduction

Uveal melanoma (UM) is the most common primary intraocular malignancy in adults with an estimated global incidence of more than 7000 cases per year [1]. At the time of diagnosis, about 2% of patients have radiologically detectable metastases [2]. Within 15 years, this proportion increases to 32–45% even with successful treatment of the eye, which is likely caused by subclinical dormant micrometastases that most frequently locate to the liver [3–5]. Once these micrometastases leave their dormant state and grow into clinically detectable lesions, few effective treatment alternatives are available and the median patient survival is one to two years [6–8].

Patients diagnosed with UM want accurate prognostic information, and patients that undergo testing confirm their desire for this information and experience lower levels of decision regret than patients who opt out - even when the result indicates a high metastatic risk [9, 10]. There are several existing methods for prognostication of this risk. Traditionally, it may be estimated by clinical features such as tumor thickness, diameter, and location, by cytogenetic aberrations such as monosomy 3, and by presence of histopathological features such as epithelioid tumor cells and vasculogenic mimicry [11–14]. More recently, sequencing of the *BAP1* gene, manual and digital assessments of immunohistochemical stains of the BAP-1 protein, and gene expression tests have shown great prognostic utility [15–19]. Samples for these tests are obtained either from enucleated eyes or by biopsy with transvitreal or transscleral techniques [20]. Although complications are rare, such procedures may require general anesthesia and lead to hemorrhage, retinal detachment, and cataract [21–24] . Furthermore, gene expression tests are associated with significant costs and may not be universally available. Less invasive and expensive tests are thus advantageous and would have obvious clinical utility. The ideal test should reflect the risk of a lethal course, be fast, inexpensive, well-tolerated, and minimally invasive [21].

Liquid biopsies based on peripheral blood samples or aqueous humor have been examined as an alternative to the prognostic tests that require access to primary tumor tissue. However, the hitherto proposed methods require repeated sampling, or provide no prognostic information beyond what is provided by a radiological examination. I.e., with very few exceptions, tests have been positive only when patients already suffer from radiologically detectable metastatic disease, or shortly before [25–44].

To address this, in this manuscript we present serUM-Px, a prognostic test based on one sample of peripheral blood obtained at the time of UM diagnosis from patients without radiologically detectable metastases. The test was developed to meet the criteria of being prognostically useful, inexpensive, and minimally invasive, and is validated in a cohort of UM patients with > 20 years follow-up.

## Methods

### Patients and serum samples

This study was approved by the Ethical Review Committee at Karolinska Institutet with an amendment from the Swedish Ethical Review Authority (Reference 2019–04297) and adhered to the tenets of the Declaration of Helsinki. The materials and methods used herein have previously been described in a preprint [45]. Eighty-tree patients that were diagnosed with posterior uveal melanoma between February 17th, 1996 and February 17th, 1999 were included. Inclusion criteria were: (1) Clinically or histopathologically diagnosed melanoma of the choroid and/or ciliary body (patients with iris melanomas were not eligible), and (2) Patient > 18 years. Exclusion criteria were: (1) Metastatic disease detectable in baseline radiological examinations, (2) Patient unable to provide informed consent, and (3) The tumor was a recurrence of a previously diagnosed and treated melanoma. Other concurrent diseases and patients’ body mass index (BMI) did not affect the eligibility for the study. After obtaining informed consent, a peripheral 10 ml venous blood sample was collected from all patients. These samples were drawn from the antecubital fossa and collected in hydrophobic plastic tubes, in which the blood was allowed to clot by leaving it undisturbed at room temperature for 15 to 30 min. The clot was then removed by centrifugation at 1500 × g for 10 min in a refrigerated centrifuge. The resulting supernatant was transferred into clean polypropylene cryotubes and stored at -80 °C within two hours of collection. All samples were preserved frozen without thawing until the analyses presented herein were performed in 2021.

At the time of diagnosis, clinicopathological data including patient age, sex, tumor location, diameter, thickness, eye laterality, ciliary body involvement, extrascleral extension, symptoms, and visual acuity were recorded in medical journals. Tumor dimensions and extent were determined with slit-lamp biomicroscopy, wide-field fundus imaging, A and B-scan ultrasonography and ultrasound biomicroscopy, as needed. Patients were then treated by either plaque brachytherapy or enucleation, depending on tumor size and location at the discretion of the patient and the attending ocular oncologist. Within one to three weeks from diagnosis (but before inclusion in the study, as information about metastatic disease was required to assess an exclusion criterion), all patients underwent radiological examinations of the thorax and abdomen, typically with contrast-enhanced computed tomography (CT).

### Follow-up

After diagnosis, screening for metastases by ultrasonography of the liver or CT of the abdomen was repeated semi-annually for a minimum of five years. Thereafter, radiological exams were not performed routinely, but when motivated by patients’ symptoms, palpable masses, deteriorating health, or by abnormal blood tests. Ocular exams were scheduled at one, three, six, and 12 months and then annually for the remainder of a patient’s life. Data on the date of radiological detection of metastases, date of death, and cause of death were obtained from patients’ medical journals. All radiologically detected metastases were biopsied for histological confirmation, and the date of histological confirmation was used as the time of event in analyses of metastasis-free survival. Follow-up data were complete even for patients that resided outside the Stockholm area, as their medical journals were available either via digitalized systems or at request by post. This data included results of radiological examinations and autopsies of patients dying from other causes than metastatic UM.

### Protein profiling and ELISA

Serum total protein content was assessed for each patient by a Bradford assay. The sample concentration (µg/µL) was determined using a calibration curve prepared with standard bovine serum albumin (BSA, Sigma-Aldrich Corp., St. Louis, MO, USA) dilutions (µg/µL). Two µl of each BSA dilution and serum sample were mixed with 200 µL of diluted Protein assay dye reagent (Bio-Rad Laboratories Hercules, CA, USA) in a 96-well plate. The absorbance was measured at 595 nm.

Serum levels of 84 cancer-related proteins were assayed by Proteome Profiler Human XL Oncology Array (BioTechne Corp., Abingdon, UK; cat. no. ARY026) in pooled serum samples from metastatic versus non-metastatic patients, as described below. A complete list of the 84 evaluated proteins is provided in **Supplementary Table** 1. The blots were developed using ECL max chemiluminescent reagent and the images were acquired by ChemiDoc XRS^+^ (both Bio-Rad Laboratories). Protein expression was determined by optical density (OD) of the dot blots corrected with the three positive controls, as recommended by the manufacturer, using Image Lab 3.0 software (Bio-Rad Laboratories). Data are presented on a log2 scatter plot (GraphPad Prism software, San Diego, CA, USA) with an interval of 2-fold difference.

Five selected proteins (selection process described in Statistical Methods section) were then quantified from single-patient serum samples by enzyme-linked immunoassay (ELISA) kits for human progranulin (cat.no. ab252364), human delta-like canonical notch ligand (DLL1) (cat.no. ab193698), human leptin (cat.no. ab179884), human osteopontin (cat.no. ab269374), human tenascin C (cat.no. ab213831), all from Abcam (Cambridge, UK). Data are presented as protein concentration (ng/ml). Each serum sample was diluted 1:10 in the sample diluent buffer prior to use, and aliquoted in a 96 well-plate. The plate was incubated at 37**°**C for 90 min. Subsequently, the plate was incubated with biotinylated antibody working solution at 37**°**C for 60 min, and then washed 3 times with 0.01 M phosphate buffered saline (PBS). Avidin/Biotin complex (ABC) working solution was added to each well, and the plate incubated at 37**°**C for 30 min, followed by 5 washes with 0.01 M PBS. 90 µL of 3,3’,5,5’-Tetramethylbenzidine (TMB) color developing agent was added to each well and incubated at 37**°**C 20 min. Lastly, 100 µL of TMB stop solution was added and the absorbance was read ta 450 nm in a microplate reader. Patients were excluded at this stage if the polypropylene cryotube containing their serum sample was visually damaged, if the serum was visually turbid or cloudy after thawing, or if the signal was oversaturated. All ELISA arrays had an internal standard control protein as supplied by the manufacturer. Known diluitons of the control protein were used as standard calibration curve for each array. Of the 65 patients originally randomized to the validation cohort (83 minus 18 patients in the training cohort), 9 were excluded for poor sample quality (six osteopontin and three leptin with oversaturated signals without readable concentrations, respectively).

### Immunohistochemistry

Metastatic risk categories according to our serum-based prognostic test were correlated to BAP-1 expression in all tumors from patients that underwent primary enucleation (*n* = 12). The formalin-fixed and paraffin-embedded (FFPE) eyes were collected from the St. Erik Ophthalmic Pathology Laboratory. Each eye was cut into a 4 μm section, pretreated in ethylenediaminetetraacetic acid (EDTA) buffer at pH 9.0 for 20 min, and incubated with mouse monoclonal antibodies against BAP-1 at dilution 1:40 (clone C-4; Santa Cruz Biotechnology; Cat# sc-28,383, RRID:AB_626723) and a red chromogen secondary antibody kit (Leica Biosystems, Nußloch, Baden-Wurttemberg, Germany), and finally counterstained with hematoxylin and rinsed with deionized water. The deparaffinization, pretreatment, primary staining, secondary staining, and counter-staining steps were run in a Bond III automated IHC/ ISH stainer (Leica, Wetzlar, Germany). The dilutions had been gradually titrated until optimal staining was achieved, according to manual control. The level of nuclear BAP-1 expression (nBAP-1) was assessed by GS according to a previously described method [18]. For a tumor to be classified as BAP-1 positive, at least 33% of tumor cell nuclei had to be positively stained, and accumulation of chromogen in nucleoli or cytoplasms did not suffice [46]. After discovery of the prognostic role of serum osteopontin in our cohorts, we also stained the 12 primary enucleated tumors with mouse monoclonal antibodies against osteopontin at dilution 1:200 (clone 7C5H12; ThermoFisher Scientific Inc.; Cat# MA5-17180, RRID: AB_2538651). All BAP-1 and osteopontin-stained glass slides were digitally scanned at ×400 (Ocus 40, Grundium Oy, Tampere, Finland). Primary tumor osteopontin expression levels were measured with QuPath Bioimage analysis v. 0.3.2 [47]. One positive and one negative cell, as manually selected by a pathologist (GS), was calibrated in each digitally scanned tissue section. This adjusts for differences in general staining intensity and color nuance between different slides. A polygon was then drawn around the margins of the tumor. Areas with inflammatory infiltrates, bleeding, heavy pigmentation, necrosis, poor fixation, uneven staining, and artefactual folding were excluded. The median optical density (OD) level of the osteopontin stain was then measured in each tumor with QuPath’s intensity computation feature (pixel size 2 μm, tile diameter 25 μm, Haralick distance 1, Haralick number of bins 32).

### Statistical methods

**Fig. 1:**
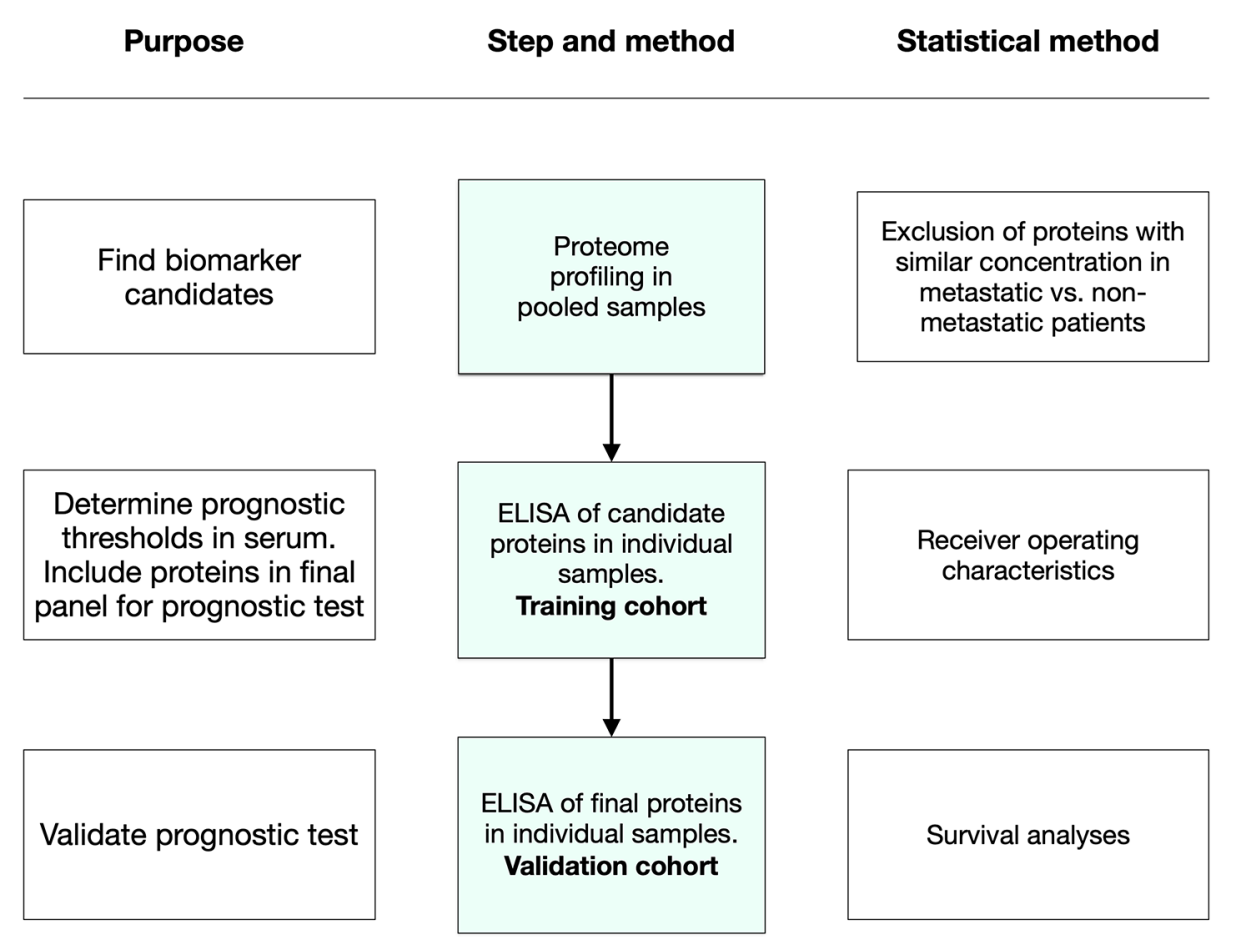
Development of serUM-Px: In step one, biomarker candidates were identified with serum protein profiling of 84 different cancer-related proteins in a pooled sample of serum from randomly selected patients that later developed metastases and patients that did not. In step two, ELISA of identified candidates from step one was then performed in individual samples in a training cohort. The thresholds for classification into metastatic risk categories were determined with receiver operating characteristics. In step three, the final serUM-Px prognostic test was validated in an independent cohort

*P* < 0.05 were considered statistically significant, all *P* values being two-sided. For tests of continuous variables that did not deviate significantly from a normal distribution (Shapiro–Wilk test *P* > 0.05) Student’s *t*-tests were used. For non-parametrical data, Mann–Whitney *U* tests were used. For comparisons of continuous variables across three categories or more, we used one-way ANOVA. In comparisons of two categorical variables, we used contingency tables and Pearson chi-square (χ^2^) tests (if all fields had a sample of > 5) or Fisher’s exact tests (if any field had a sample of < 5). In comparison of categorical and ordinal variables, the Kruskal-Wallis test was used. The inter-method agreement between primary tumor BAP-1 expression and serUM-Px metastatic risk category was assessed with Cohen’s kappa coefficient (κ). Patients’ BMI was calculated as a person’s weight in kilograms divided by their height in meters squared. The calculated BMI was used to stratify patients into four categories: Class I, underweight (BMI < 18.5); Class 2, normal weight (BMI ≥ 18.5 to 24.9); Class 3, overweight (≥ 25 to 29.9); and Class 4, obese (BMI ≥ 30.0), according to a classification used by the National Institutes of Health (NIH) [48]. The prognostic test was developed in three steps: Firstly, biomarker candidates were identified with protein profiling of 84 different cancer-related proteins in a pooled sample of serum from ten randomly selected patients that later developed metastases and 12 patients that did not. Secondly, ELISA of identified candidates was then performed in a training cohort. Optimal cutoffs for classification into metastatic risk categories were determined with receiver operating characteristics (ROC) in this cohort. Equal emphasis was put on sensitivity and specificity for metastasis, and the cutoff value representing the largest combined sum of sensitivity + specificity (i.e., accuracy) was chosen. The minimum requirement for a prognostically useful test was that it had an area under the curve (AUC) with a lower bound of the asymptotic 95% confidence interval (CI) of > 0.5. Thirdly, the final serUM-Px prognostic test with cutoff values established in the training cohort was then validated in an independent validation cohort (Fig. 1). The relative size of the training and validation cohort was determined after a power analysis with emphasis on how many patients would be required for survival analysis in the validation cohort. We arbitrarily assumed a metastatic incidence of ten and 40% in absence and presence of a poor prognostic marker, respectively, an alpha of 0.05 and a beta of 80%, which meant that approximately 62 patients would have to be allocated to the validation cohort. The total sample of 83 patients were therefore randomized into the training and validation cohort in a 1:3 ratio (18 patients in the training cohort, 65 patients in the validation cohort, of which 9 were excluded for poor sample quality as described above). Metastasis-free and overall survival curves were generated by the Kaplan-Meier method and the Log-rank test was applied. For comparisons of association with metastasis, multivariate Cox regression hazard ratios (HR) were calculated. Considering the risk of influence from competing risks on metastasis-free survival (i.e., death from other causes before the development of metastases), cumulative incidence function estimates from competing risks data were also plotted with the *cmprsk* package for R, and the equality of survival distributions was tested with Gray’s test for equality [49]. The overall survival rate of the included sample was contrasted to the expected survival of males and females of identical age from the general population using data from Swedish life tables with year-per-year statistics [50]. All statistical analyses were performed using IBM SPSS statistics version 27 (Armonk, NY, USA) and GraphPad Prism version 9.3.0 (San Diego, CA, USA). The Sankey diagram was generated with SankeyMATIC (https://sankeymatic.com).

## Results

### Descriptive statistics

83 patients were recruited to this study at the time of their primary UM diagnosis between February 17th, 1996, and February 17th, 1999. No patient had radiologically detectable metastases at the time of diagnosis, was pregnant or was known to have liver dysfunction. 22 patients developed metastases during follow-up and all these eventually succumbed to their disease. Forty-two patients died from other causes. All patients followed their schedule of semi-annual metastatic screening, and no patient was lost to follow-up. The median follow-up for the 19 survivors (23% of the original sample) was 22.7 years (IQR 22.3–23.0). The expected survival proportion calculated from remaining life expectancy in the general population was 29% at 22.5 years (95% CI 19 to 39%).

There were no significant differences in patient age, sex, ciliary body involvement, chief complaint at presentation, visual acuity, tumor thickness, diameter, AJCC T-category, AJCC stage, primary tumor treatment, or in the proportion of patients that developed metastases between the 18 patients randomized to the training cohort, the 56 patients randomized to the validation cohort and the 9 patients that were excluded for poor serum sample quality in later stages (as described in the validation cohort-section). The exception was tumor eye laterality, with right eyes being more common in the training cohort (chi-square *P* = 0.04, Table 1).

**Table 1:** Demographics and clinical features of study patients

	All	Training	Validation	Excluded	P*
***n***	83	18	56	9	
**Age at diagnosis, mean (SD)**	65 (12)	64 (11)	65 (13)	72 (14)	0.27
**Sex, n (%)**					0.96
Female	43 (52)	9 (50)	27 (48)	4 (44)	
Male	40 (48)	9 (50)	29 (52)	5 (56)	
**Ciliary body involvement, n (%)**					0.79
Yes	3 (4)	1 (6)	2 (4)	0 (0)	
No	80 (96)	17 (94)	54 (969	9 (100)	
**Tumor eye laterality, n (%)**					0.04
Right	49 (59)	15 (83)	28 (50)	6 (67)	
Left	34 (41)	3 (17)	28 (50)	3 (33)	
**Extrascleral extension, n (%)**					0.78
Yes	1 (1)	0 (0)	1 (2)	0 (0)	
No	82 (99)	18 (100)	55 (98)	9 (100)	
**Chief complaint at presentation, n (%)**					0.18
Shadow in visual field	10 (12)	1 (6)	8 (14)	1 (11)	
Reduced visual acuity	19 (23)	4 (22)	12 (21)	3 (33)	
Pain	1 (1)	0 (0)	0 (0)	1 (11)	
Floaters	4 (5)	1 (6)	3 (5)	0 (0)	
Photopsia	11 (13)	4 (22)	5 (9)	2 (22)	
No symptoms	38 (46)	8 (44)	28 (50)	2 (22)	
**Visual acuity at diagnosis, mean LogMAR (SD)**	0.5 (0.3)	0.5 (0.3)	0.5 (0.3)	0.6 (0.2)	0.61
**Tumor thickness at diagnosis, mean mm (SD)**	5.7 (2.6)	5.2 (2.8)	5.6 (2.5)	6.6 (2.9)	0.44
**Tumor diameter at diagnosis, mean mm (SD)**	10.6 (2.4)	11.0 (2.4)	10.6 (2.4)	11.0 (2.5)	0.80
**AJCC T-category at diagnosis, n (%)**					0.22
T1a	25 (30)	8 (44)	14 (25)	3 (33)	
T1b-d	0 (0)	0 (0)	0 (0)	0 (0)	
T2a	40 (48)	6 (33)	32 (57)	2 (22)	
T2b-d	0 (0)	0 (0)	0 (0)	0 (0)	
T3a	17 (21)	4 (22)	9 (16)	4 (44)	
T3b-d	0 (0)	0 (0)	0 (0)	0 (0)	
T4a-c	0 (0)	0 (0)	0 (0)	0 (0)	
T4e	1 (1)	0 (0)	1 (2)	0 (0)	
**AJCC stage at diagnosis, n (%)**					0.22
I	25 (30)	8 (44)	14 (25)	3 (33)	
IIA	40 (48)	6 (33)	32 (57)	2 (22)	
IIB	17 (21)	4 (22)	9 (16)	4 (44)	
IIIA	0 (0)	0 (0)	0 (0)	0 (0)	
IIIB	0 (0)	0 (0)	0 (0)	0 (0)	
IIIC	1 (1)	0 (0)	1 (2)	0 (0)	
IV	0 (0)	0 (0)	0 (0)	0 (0)	
**Primary treatment, n (%)**					0.43
Plaque brachytherapy	71 (86)	15 (83)	47 (84)	9 (100)	
Enucleation	12 (14)	3 (17)	9 (16)	0 (0)	
**Metastasis, n (%)**					0.16
Yes	22 (27)	5 (28)	17 (30)	0 (0)	
No	61 (73)	13 (72)	39 (70)	9 (100)	
**Median follow-up for survivors, years (IQR)**	22.7 (0.7)	22.6 (0.5)	22.9 (0.7)	22.4 (0.5)	0.69

SD, Standard deviation. AJCC, American Joint Committee on Cancer. IQR, Interquartile Range. *P value for difference between training, validation and excluded patients as determined with one-way ANOVA for continuous variables and chi-square tests for categorical variables.

### Serum protein profiling

In order to assess prognostically relevant protein biomarkers, we first performed serum protein profiling by screening 84 different cancer-related proteins in a pooled sample of serum from 10 randomly selected patients that later developed metastases and 12 patients that did not. Proteins were selected for further analysis if they, (1) deviated between the pool of metastatic and non-metastatic patients, and (2) did not have a similar biological function as another protein with higher deviation between the groups. Based on these factors, 5 proteins were included for further analysis: leptin, osteopontin, progranulin, tenascin C and DLL1 (**Supplementary Fig.** 1). Epidermal growth factor receptor (EGF R), Receptor tyrosine-protein kinase erbB-3 (ErbB3/HER3), metalloproteinase 3 (MMP3) had higher concentrations in the pooled sample from metastatic patients but were not selected for overlapping functions with proteins with higher deviation (i.e., EGF R, ErbB3/HER3 being growth factor receptors vs. progranulin which is a secreted growth factor; and MMP3 vs. osteopontin which both are involved in wound healing and breakdown and remodeling of extracellular matrix and bone) [51].

### Training cohort

We next confirmed our findings in an independent cohort by performing an ELISA of each of the 5 selected proteins in the 18 patients randomized to the training cohort. None were excluded before further analysis due to poor sample quality.

**Fig. 2:**
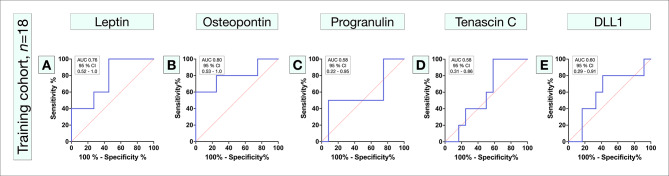
Receiver operating characteristics of five proteins that qualified from protein profiling in the training cohort. (**A**) Leptin, and (**B**) osteopontin met the minimum requirement for a prognostically useful test according to our definition (i.e., lower bound of AUC 95% CI > 0.5). (**C**) Progranulin, (**D**) tenascin C, and (**E**) DLL1 did not meet the minimum requirement. AUC, area under the curve. CI, confidence interval

In ROC, low leptin levels (AUC 0.76, 95% CI 0.52 to 1.0, Fig. 2A), and high osteopontin levels (AUC 0.80, 95% CI 0.53 to 1.0, Fig. 2B) met the minimum requirement for a prognostically useful test according to our definition (i.e., lower bound of AUC 95% CI > 0.5). Serum leptin levels are known to correlate to patient sex, and in our data, females had significantly higher serum leptin levels (mean 22 vs. 11 ng/mL, Mann–Whitney *U P* < 0.001). Based on ROC, the cutoffs with highest accuracy for metastasis was < 5 ng/ml and < 15 ng/mL for leptin levels in males and females, respectively, and > 5 ng/mL for osteopontin levels. Progranulin (AUC 0.58, 95% CI 0.22 to 0.95, cutoff 44 ng/ml, Fig. 2C), tenascin C (AUC 0.58, 95% CI 0.31 to 0.86, cutoff 9 ng/ml, Fig. 2D) and DLL1 (AUC 0.60, 95% CI 0.29 to 0.91, cutoff 6 ng/ml, Fig. 2E) did not meet the minimum requirement.

Based on these results, progranulin, tenascin C, and DLL1 were eliminated, while leptin and osteopontin were included in the final prognostic test (serUM-Px). This panel was constructed so that patients could be assigned to one of three metastatic risk categories: low; intermediate; or high (Table 2). As expected from the power analysis, patients in the very limited training cohort (*n* = 18) did not have shorter metastasis-free survival with increasing metastatic risk category (Log-rank *P* for trend = 0.09, **Supplementary Fig.** 1).

**Table 2:** SerUM-Px test classification

Metastatic risk category	Definition
Low	Serum Leptin concentration high, *and* serum Osteopontin concentration low
Intermediate	Serum Leptin concentration low, *or* serum Osteopontin concentration high
High	Serum Leptin concentration low, *and* serum Osteopontin concentration high

Low Leptin defined as < 5 ng/ml (men) and < 15 ng/ml (women). High Osteopontin defined as > 5 ng/ml

### Validation cohort

**Fig. 3:**
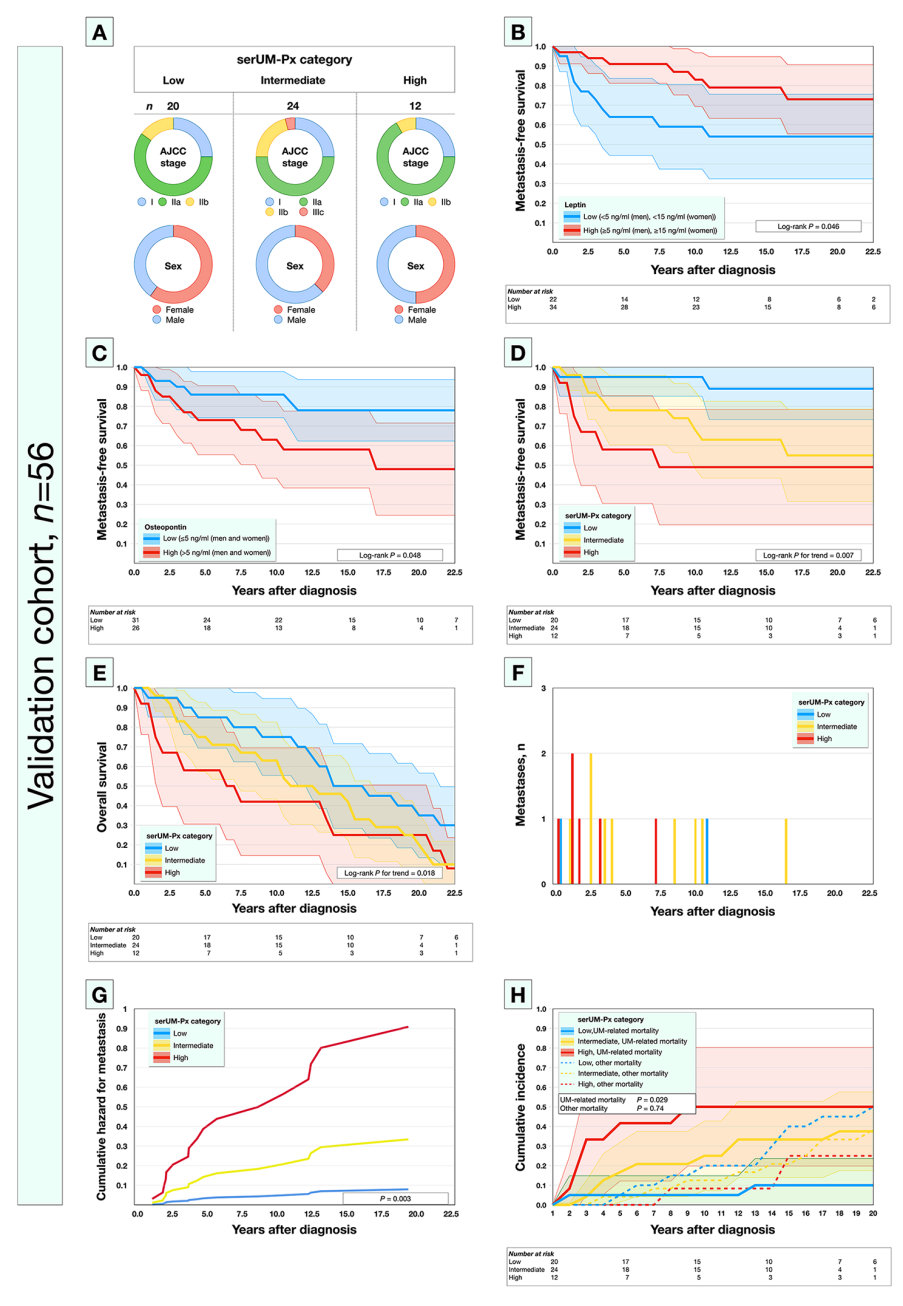
Survival analyses in the validation cohort (*n* = 56). (A) Circle diagrams, distribution of AJCC stage (Fisher’s exact *P* = 0.86) and patient sex (*P* = 0.33) over serUM-Px metastatic risk categories. (B) Patients with low serum leptin, and (C) high serum osteopontin at diagnosis had shorter metastasis-free survival. (D) Patients had shorter metastasis-free, and (E) overall survival with increasing serUM-Px metastatic risk category. (F) Metastases appeared up to 16 years after diagnosis, with 65% (11 of 17), 76% (13 of 17), and 94% (16 of 17) of metastases occurring during the first five, ten, and 15 years after diagnosis, respectively. (G) In multivariate Cox regression with patient sex, tumor diameter and serUM-Px metastatic risk category as covariates, serUM-Px was an independent predictor of metastasis (hazard ratio 3.2, 95% CI 1.5 to 6.9). (H) Cumulative incidence of UM related mortality in competing risk analysis. Patients had significantly greater incidence of UM-related mortality with increasing serUM-Px metastatic risk category, but not in death from other causes. For definition of the serUM-Px categories, see Table 2. Colored areas represent 95% confidence intervals

Patient sex and age at diagnosis, tumor size, AJCC stage, primary treatment modality, median follow-up, and median time to metastasis were evenly distributed among the 56 patients in three metastatic risk categories. In ROC, leptin and osteopontin achieved an AUC of 0.69 and 0.63, respectively (**Supplementary Fig.** 1). Patients in the three metastatic risk categories were similar with regard to widely recognized clinical risk factors of importance, and any survival differences between the groups could not be explained by differences in these (Table 3; Fig. 3A).

**Table 3:** Clinicopathological features across metastatic risk categories in validation cohort

	Low	Intermediate	High	*P* ^b^	
***n*** **=**	20	24	12		
**Mean age at diagnosis, years (SD)**	61 (13)	67 (11)	66 (14)	0.32	
**Sex, n (%)**				0.33	
Female	12 (60)	9 (38)	6 (50)		
Male	8 (40)	15 (63)	6 (50)		
**Mean tumor thickness, mm (SD)**	5.0 (2.2)	5.9 (2.7)	6.2 (2.6)	0.37	
**Mean tumor diameter, mm (SD)**	10.4 (2.7)	10.9 (2.4)	10.4 (1.8)	0.72	
**AJCC stage at diagnosis, n (%)**				0.86	
I	5 (25)	6 (25)	3 (25)		
IIA	12 (60)	12 (50)	8 (67)		
IIB	3 (15)	5 (21)	1 (8)		
IIIA	0 (0)	0 (0)	0 (0)		
IIIB	0 (0)	0 (0)	0 (0)		
IIIC	0 (0)	1 (4)	0 (0)		
IV	0 (0)	0 (0)	0 (0)		
**Primary treatment, n (%)**				0.11	
Plaque brachytherapy	19 (95)	20 (83)	8 (67)		
Enucleation	1 (5)	4 (17)	4 (33)		
**Median follow-up**^**a**^, **years (IQR)**	23.1 (0.3)	20.5 (1.5)	-	0.07	
**Median time to metastasis, years (IQR)**	6.1 (5.2)	4.4 (7.8)	1.4 (1.6)	0.23	

SD, Standard deviation. IQR, Interquartile range. ^a^For survivors. Six patients in the low metastatic risk category were alive at the end of follow-up, two patients in the intermediate metastatic risk category and zero patients in the high metastatic risk category. ^b^One-Way ANOVA for continuous variables in three groups, Mann–Whitney *U* test for two groups, chi-square test for categorical variables

Patients with low serum leptin levels at diagnosis had shorter metastasis-free survival (Log-rank *P* = 0.046, Fig. 3B). Similarly, patients with high osteopontin levels had shorter metastasis-free survival (*P* = 0.048, Fig. 3C). Patients also had shorter metastasis-free survival with increasing metastatic risk category (Log-rank *P* for trend = 0.007, Fig. 3D). Twenty patients in the low metastatic risk category had a five, ten, 15, and 20-year metastasis-free survival of 95, 95, 89, and 89%, respectively. 24 patients in the intermediate metastatic risk category had a five, ten, 15, and 20-year metastasis-free survival of 78, 68, 63, and 55%, respectively. 12 patients in the high metastatic risk category had a five, ten, and 20-year metastasis-free survival of 58, 49, 49. and 49%, respectively. Patients also had worse overall survival with increasing metastatic risk category (*P* = 0.018, Fig. 3E).

Metastases appeared up to 16 years after diagnosis, with 65% (11 of 17), 76% (13 of 17) and 94% (16 of 17) of metastases occurring during the first five, ten and 15 years after diagnosis, respectively (Fig. 3F).

In univariate Cox regression, serUM-Px and tumor diameter were significant predictors of metastasis (HR 2.4 per increasing metastatic risk category, 95% CI 1.2 to 4.6, and HR 1.4 per increasing mm, 95% CI 1.1 to 1.7). In multivariate regression with tumor diameter and serUM-Px as covariates, both retained their significance (HR 1.4, 95% CI 1.2 to 1.8, and HR 3.1, 95% CI 1.5 to 6.8, respectively). In multivariate regression with both of tumor diameter and patient sex as covariates, tumor diameter and serUM-Px still retained their significance (HR 1.5, 95% CI 1.2 to 1.8, and HR 3.2, 95% CI 1.5 to 6.9, respectively, Table 4; Fig. 3G).

**Table 4:** Cox regressions, hazard for metastasis

Univariate	B	S.E.	Wald	*P*	Exp(B)	95% CI lower	95% CI upper
Patient age at diagnosis^a^	-0.1	0.2	0.6	0.44	0.9	0.6	1.2
Patient sex, male vs. female	-0.1	0.5	0.1	0.84	0.9	0.3	2.3
Tumor diameter, mm^b^	0.3	0.1	8.6	0.003	1.4	1.1	1.7
Tumor thickness, mm^b^	0.1	0.1	0.5	0.46	1.1	0.9	1.3
SerUM-Px^c^	0.9	0.3	6.6	0.010	2.4	1.2	4.6
**Multivariate**							
Tumor diameter, mm^b^	0.4	0.1	11.1	0.001	1.4	1.2	1.8
SerUM-Px^c^	1.1	0.4	8.5	0.004	3.1	1.5	6.8
**Multivariate**							
Patient sex, male vs. female	0.5	0.5	0.9	0.34	1.7	0.6	5.1
Tumor diameter, mm^b^	0.4	0.1	11.0	0.001	1.5	1.2	1.9
SerUM-Px^c^	1.2	0.4	9.1	0.003	3.2	1.5	6.9
^a^Per increasing decade. ^b^Per increasing mm. ^c^Per increasing metastatic risk category.							

In cumulative incidence function estimates from competing risks data, patients had a significantly greater incidence of UM-related mortality with increasing serUM-Px metastatic risk category (Gray’s test for equality *P* = 0.029) but not of mortality from other causes (*P* = 0.74, Fig. 3H).

### Primary tumor BAP-1 expression versus metastatic risk category

**Fig. 4:**
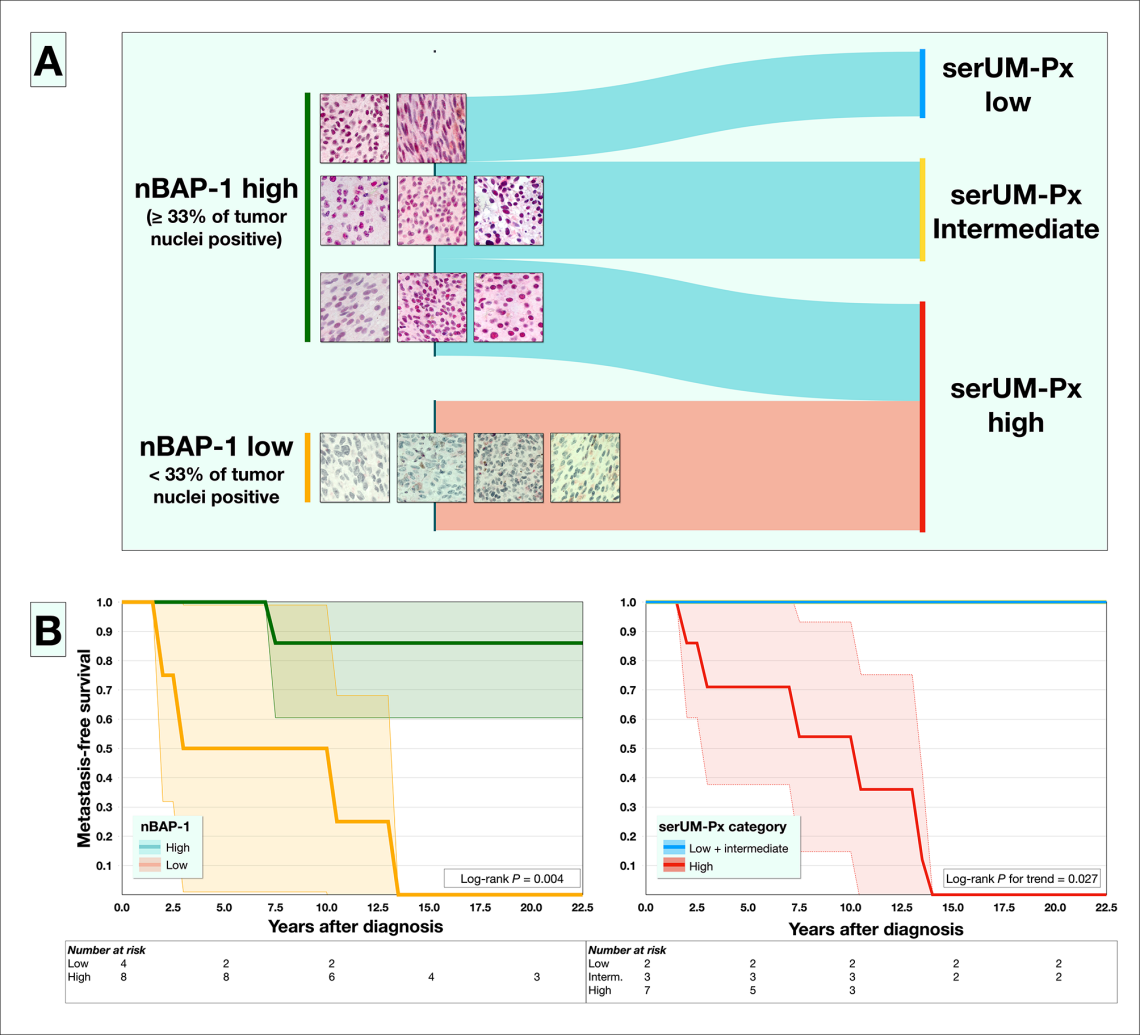
Nuclear BAP-1 expression (nBAP-1) versus serUM-Px metastatic risk category. Of 12 available primary enucleations, four (33%) had low nuclear BAP-1 expression (associated with high metastatic risk) and eight (67%) had high expression (associated with low metastatic risk). (A) Sankey diagram showing the flow of classifications between nBAP-1 and serUM-Px category. (B) Metastasis-free survival curves for the same 12 patients, according to nBAP-1 status (left) and serUM-Px category (right). There were no metastatic events in the low and intermediate metastatic risk categories. For definition of the serUM-Px categories, see Table 2. Colored areas represent 95% confidence intervals

Loss of expression of BAP-1 in a majority of tumor cell nuclei (low nBAP-1) is a strong marker for poor patient prognosis in UM. Of the 12 patients that underwent primary enucleation, four (33%) had tumors with low nBAP-1 (associated with high metastatic risk) and eight (67%) had tumors with high expression (associated with low metastatic risk). Tumors with high nBAP-1 expression were enucleated from patients in all three serUM-Px metastatic risk categories, but all four tumors with low nBAP-1 were enucleated from patients in the high metastatic risk category (Fig. 4A, **supplementary Table** 1).

There was a moderate inter-method agreement between nBAP-1 and serUM-Px category dichotomized as high versus low or intermediate (κ = 0.53, *P* = 0.038). The four patients with tumors that had low nBAP-1 had significantly shorter metastasis-free survival (Log-rank *P* = 0.004, Fig. 4B). Similarly, seven patients in the serUM-Px high metastatic risk category had significantly shorter metastasis-free survival. There were no metastatic events in the intermediate and low metastatic risk categories (Log-rank *P* for trend = 0.027).

### Primary tumor osteopontin expression

**Fig. 5:**
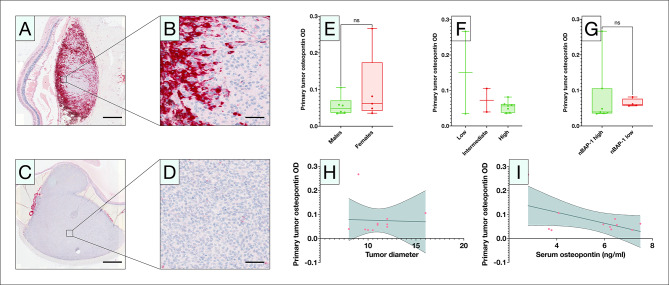
Primary tumor osteopontin expression. A and B) In immunohistochemical assessments, osteopontin (red) was primarily expressed in macrophages, granulocytes, lymphocytes, and in the extracellular matrix, but also in tumor cells in the peripheral regions of tumors. C and D) Other tumors had no visible expression. E) Primary tumor osteopontin expression levels were not associated with patient sex, F) serUM-Px category, or G) nBAP-1. Similarly, in linear regressions, primary tumor osteopontin expression levels were not associated with H) primary tumor diameter (R^2^ = 0.001, *P* = 0.91), or I) serum osteopontin levels (R^2^ = 0.30, *P* = 0.08). For definition of the serUM-Px categories, see Table 2. OD, optical density. nBAP-1, nuclear BAP-1 expression level. Ns, non-significant. Scale bars: Overview 1 mm, insert 100 μm

We also used digital bioimage analysis to measure primary tumor osteopontin expression levels in order to assess its correlation with serum osteopontin levels. In histological assessments, osteopontin was primarily expressed in macrophages, granulocytes, lymphocytes, and in the extracellular matrix (Fig. 5A and D). Primary tumor osteopontin expression levels were similar between males and females (Mann–Whitney *U P* = 0.43, Fig. 5E), between serUM-Px categories (Kruskal-Wallis *P* = 0.92, Fig. 5F), and between primary tumors with low and high nBAP-1 expression (Mann–Whitney *U P* = 0.32, Fig. 5G). In linear regressions, primary tumor median, minimum or maximum expression levels of osteopontin had no correlation with serum osteopontin levels (R^2^ 0.13 to 0.30, *P* = 0.08 to 0.29, Fig. 5H) or to primary tumor diameter (R^2^ = 0.001, *P* = 0.91, Fig. 5I). Further, in univariate Cox regression, primary tumor osteopontin expression levels were not a significant predictor of metastasis (HR 0.1 per increasing OD unit, 95% CI < 0.001 to 332,452).

### Serum leptin levels versus body mass index

As reported above, females had significantly higher serum leptin levels. By examination of archived medical records, we could also obtain data on the patients’ weight and height at the time of UM diagnosis for 23 of the patients, which allowed for calculation of body mass index (BMI). In linear regression, leptin levels had a positive correlation with BMI (standardized slope coefficient 0.79, *P* = 0.041, **Supplementary Fig.** 1). 18% of the variation in serum leptin could be explained by BMI (R^2^ 0.18). In univariate Cox regressions, low leptin levels (5 ng/ml and < 15 ng/ml for males and females, HR 2.7, 95% CI 1.2 to 6.3) were associated with metastasis, but not BMI as a continuous variable (HR 0.9 per increased integer, 95% CI 9.8 to 1.1) or as class 1 to 4 (HR 0.8 per increased class, 95% CI 0.3 to 1.7). In multivariate regression, none of patient sex, leptin levels, or BMI was an independent predictor of metastasis (**Supplementary Table** 1).

## Discussion

In this study, we have developed a prognostic test for UM based on a single peripheral blood sample that is obtained at the time of diagnosis. In a three-step procedure, we identified the strongest candidates from a panel of 84 proteins, established threshold levels in a training cohort and then validated the final serUM-Px test in a separate sample. We demonstrate that a low, intermediate, and high metastatic risk category can be defined based on serum leptin and osteopontin levels, and that patients have shorter metastasis-free- and overall survival as well as a greater incidence of UM-related mortality with each increasing metastatic risk category.

Liquid biopsies have recently been investigated as an alternative approach to detect and monitor disease progression for patients with UM [25–44]. Liquid biopsy involves the sampling of tumor-derived molecules in body fluids such as blood or aqueous humor [21]. This technique includes various components such as circulating tumor cells (CTCs), circulating tumor DNA (ctDNA), cell-free microRNAs, as well as tumor-derived extracellular vesicles (EVs) [21]. Some of these techniques have shown promise in UM, including cell free-micro RNAs [38]. Recently, plasma levels of blood-based B-cell activating factor (BAFF), growth differentiation factor-15 (GDF-15), and osteopontin were combined into a panel that could distinguish patients with and without radiologically detectable metastases [52]. I.e., this test added no information beyond what is provided by a radiological examination. In a subgroup of 24 patients however, plasma levels of BAFF and GDF-15 were observed to increase significantly in the period 0 to 6 months before clinical detection of metastases. Other liquid biopsies have not shown any significant correlations with prognosis, such as ctDNA which seems to be more suitable for the use of monitoring treatment response and disease course rather than for prediction of metastases [21].

Two other previous studies found a significant difference in plasma levels of osteopontin between patients with and without detectable metastases [40, 41]. In one of these, tumor marker levels including serum osteopontin increased before the existence of radiologically detectable metastases [40]. Osteopontin is a 314-amino acid phosphoglycoprotein that is a component of the noncollagenous bone matrix [41]. This protein has been described in the role of diverse physiological roles such as chemotaxis, cell migration and adhesion, angiogenesis, apoptosis, cell-extracellular matrix interactions, and immune regulation [53]. Osteopontin actively promotes the tumorigenic phenotype and contributes to metastatic spread.[41] Elevated serum levels of osteopontin have been described in patients with advanced or metastatic cancer [41]. Recently, increased osteopontin levels have been observed in patients with metastatic UM, which correlates with our results [54–56]. We found no correlation between primary tumor and serum levels of osteopontin, and primary tumor osteopontin expression levels did not correlate with patients’ prognosis. This could indicate that the source of high serum levels of osteopontin is related to micrometastatic disease and extracellular matrix remodeling at the metastatic niche, rather than leakage of osteopontin to the blood stream from the primary tumor.

The other variable in the prognostic test – leptin – has been described in various types of tumor cells, including breast, prostate, colon and endometrium where leptin has been implicated as a growth factor for these cancers [57–61]. Leptin does not only play a role in food intake and energy balance but also functions as a pro-inflammatory adipokine with a broad range of activities including cytokine production, cellular immunity, and angiogenesis [62–64]. Leptin may promote tumor growth by signaling through normal endocrine pathways: Physiologic binding of leptin to its receptors on hypothalamic neurons leads to Thyrotropin-releasing hormone (TRH) production by these cells [62]. Ellerhorst et al. have shown that melanoma cells express TRH and that TRH induces proliferation of these cells, which raises the possibility of leptin as an inducer of melanoma TRH production and secretion, accounting in part for its growth-promoting effects [65]. It remains unclear why leptin seems to have an opposite role in UM, with increased levels associated with a protective effect. Other than increased levels of leptin, a high BMI is also associated with lower plasma levels of adiponectin [66, 67]. Low adiponectin levels have been shown to increase the risk for UM metastases [68]. Further, the liver synthesized growth factors IGF-1 and HGF/SF are affected by levels of exercise, stress, nutrition and BMI, and may contribute to metastatic progression and tumor cell homing to the liver [69, 70].

Considering that most patients diagnosed with UM desire prognostic information and that most current testing alternatives entail an invasive procedure unless the tumor eye is enucleated, liquid biopsies based on peripheral blood samples are an attractive alternative. We suggest that this newly developed test may be seen as an alternative to FNABs and transvitreal biopsies. serUM-Px has the benefit of being a test that reflects the risk of lethal course, is relatively inexpensive, minimally invasive, and has a low risk profile regarding complications. Consequently, prognostic testing can be made available for all UM patients, regardless of treatment modality.

Other strengths of this study include the complete control of patients’ follow-up. We had access to detailed data regarding the tumor and patient characteristics, as well as survival data from clinical records that were accessible regardless of where in the country the patient resides, which enabled robust correlation to the outcome where no patient was lost to follow-up. Further, our test predicted metastatic disease many years before macrometastases developed, whereas most other similar tests have relied on repeated sampling to reveal macrometastases at the time of or just before they become radiologically detectable [21]. Another of the foremost strengths of this study is simultaneously one of its considerable limitations; the > 20-year storage of the serum samples at -80 °C allowed for long follow-up. However, no fresh samples were included. Even though previous studies indicate that serum samples can be stored deep-frozen even for decades without protein degradation, the protein concentrations observed herein do not necessarily reflect concentrations in fresh samples [71–73].

### Limitations of the study

This study has several other limitations. The results were based on a relatively small cohort of patients with moderately few metastatic events. The latter is likely a result of inclusion of a cohort with quite small tumors. Tumor size is strongly associated with virtually all other prognostic factors in UM, including ciliary body involvement, *BAP1* mutation, gene expression class 2, monosomy 3, tumor cell type, and patient age [74–76]. Several of these factors were not included in our data and we cannot assess their correlation with serUM-Px. The serUM-Px metastatic risk categories were not associated with primary tumor BAP-1 expression, which is a well-established strong prognostic marker [77]. As this correlation was examined in an even smaller sample of 12 tumors, we suspect that the non-significant correlation (*P* = 0.056) may represent a type II error, which should be investigated in a larger cohort. The small sample size also increases the risk that we eliminated candidate proteins that would have contributed with prognostic information in a larger sample. Further, serum leptin levels have a diurnal variation that follows the circadian rhythm with peak levels at night [78]. Even though all of our blood samples were taken in the daytime, some patients may have been classified differently if their blood sample had been drawn at a different time of the day [79].

## Conclusions

We have developed a novel prognostic test based on a single peripheral venous blood sample at the time of UM diagnosis. This test stratifies patients into metastatic risk categories and predicts metastases up to many years in advance in an independent validation cohort with long follow-up. Patients in the low, intermediate, and high metastatic risk category have a 10-year metastasis-free survival of 95, 68 and 49%, respectively which can be used to tailor follow-up intervals for metastatic screening, and selection criteria for clinical trials. Further prospective validation of the serUM-Px test may contribute to the implementation of non-invasive prognostic testing in UM.

## Electronic supplementary material

Below is the link to the electronic supplementary material.

Supplementary Material 1

## Data Availability

The datasets supporting the conclusions of this article are included within the article and its additional files.

## Abbreviations

AJCC: American Joint Committee on Cancer
AUC: area under the curve
BAP-1: BRCA1 Associated Protein-1
BSA: bovine serum albumin
CT: computed tomography
CTCs: circulating tumor cells
ctDNA: circulating tumor DNA
DLL1: human Delta-like canonical Notch ligand
EDTA: ethylenediaminetetraacetic acid
EGF R: Epidermal growth factor receptor
ELISA: enzyme-linked immunoassay
ErbB3/HER3: Receptor tyrosine-protein kinase erbB-3
FFPE: formalin-fixed and paraffin-embedded
HR: hazard ratio
IHC: immunohistochemistry
ISH: In situ hybridization
IQR: interquartile range
MMP3: metalloproteinase 3
OD: optical density
ROC: receiver operating characteristics
SD: standard deviation
UM: uveal melanoma
