# Supplementary material for: A serum protein signature at the time of Uveal Melanoma diagnosis predicts long-term patient survival
Source: BMC Cancer. 2023 Mar 27;23:277. doi: 10.1186/s12885-023-10757-x (PMC10044715; doi:10.1186/s12885-023-10757-x)
Supplement: Supplementary file 1 — Supplementary Material 1 [file 12885_2023_10757_MOESM1_ESM.pdf]

Supplementary material to:

A Serum Protein Signature at the Time of Uveal Melanoma Diagnosis Predicts Long-Term Patient Survival

Christina Herrspiegel, M.D.†[1,2], Flavia Plastino, M.Sc.†[2], Emma Lardner BMS.[1], Stefan Seregard, M.D., Ph.D.[1,2], Pete A. Williams, Ph.D.[1,2], Helder André, Pharm.D., Ph.D.[2], Gustav Stålhammar*, M.D., Ph.D.[1,2]

[1]St. Erik Eye Hospital, Stockholm, Sweden.
[2]Department of Clinical Neuroscience, Division of Eye and Vision, Karolinska Institutet, Stockholm, Sweden.
†Co-first authors

Supplementary table 1. List of proteins examined in evaluation of biomarker candidates

Protein no.	Protein name	Entrez Gene ID	Alternate Nomenclature
1	α-Fetoprotein	174	AFP, DSCAM2
2	Amphiregulin	374	AREG
3	Angiopoietin-1	284	ANGPT1
4	Angiopoietin-like 4	51129	ANGPTL4
5	ENPP-2/Autotaxin	5168	ATX, Lysophosphatidic Acid, NPP2, PDNP2
6	Axl	558	Ark, Ufo
7	BCL-x	598	BCL2L1
8	CA125/MUC16	94025	MUC16
9	E-Cadherin	999	Arc-1, CAD1, Cadherin-1, CD324, CDH1, Cell-CAM120/80, ECAD, L-CAM
10	VE-Cadherin	1003	Cadherin-5, CD144, CDH5
11	CapG	822	AFCP
12	Carbonic Anhydrase IX	768	CA9, G250, MN, RCC
13	Cathepsin B	1508	CTSB
14	Cathepsin D	1509	CTSD
15	Cathepsin S	1520	CTSS
16	CEACAM-5	1048	CD66e, CEA
17	Decorin	1634	DCN, DSPG2, PG-II, PSG2, SLRR1B
18	Dkk-1	22943	Dickkopf-1
19	DLL1	28514	Delta 1
20	EGF R/ErbB1	1956	ErbB, ErbB1, HER-1
21	Endoglin/CD105	2022	CD105, ENG
22	Endostatin	80781	COL18A1
23	Enolase 2	2026	ENO2; γ-Enolase; NSE
24	eNOS	4846	NOS3
25	EpCAM/TROP1	4072	17-1A, CD326, GA733-2, gp40, KS1/4, M4S1, TACSTD1
26	ERα/NR3A1	2099	ESR1, NR3A1
27	ErbB2	2064	CD340, HER2, Neu Oncogene, NGL, TKR1
28	ErbB3/Her3	2065	HER3
29	ErbB4	2066	HER4
30	FGF basic	2247	FGF2, FGF-2, FGF2AS, GFG1, HBGH-2, NUDT6, Prostatropin
31	FoxC2	2303	Fkh14, LD, MFH1
32	FoxO1/FKHR	2308	FKH1, FKHR
33	Galectin-3	3958	AGE-R3, CBP35, GAL3, L29, LGALS3, Mac-2
34	GM-CSF	1437	CSF2
35	CG α/β (HCG)	1081 (α)/1082 (β)	CGB, CGB3, Choriogonadotropin
36	HGF R/c-Met	4233	MET
37	HIF-1α	3091	HIF1A
38	HNF-3β	3170	FoxA2
39	HO-1/HMOX1	3162	HSP32
40	ICAM-1/CD54	3383	
41	IL-2 Rα	3559	CD25, IL2RA
42	IL-6	3569	BSF-2, IFN-β2, MGI-2A
43	CXCL8/IL-8	3576	GCP1, IL8, LAI, MDNCF, NAP1, NCF, TCF, TSG1
44	IL-18 BPa	10068	IL18BP
45	Kallikrein 3/PSA	354	KLK3
46	Kallikrein 5	25818	KLK5, KLK-L2, SCTE
47	Kallikrien 6	5653	KLK6, Neurosin, Protease M, PRSS18, PRSS9, SP59, Zyme
48	Leptin	3952	LEP, OB
49	Lumican	4060	LDC, LUM, SLRR2D
50	CCL2/MCP-1	6347	MCAF
51	CCL8/MCP-2	6355	
52	CCL7/MCP-3	6354	MARC
53	M-CSF	1435	CSF1, CSF-1
54	Mesothelin	10232	CAK1, MPF, MSLN, SMR
55	CCL3/MIP-1α	6348/6351	LD78a; MIP-1 alpha
56	CCL20/MIP-3α	6364	exodus-1; LARC; MIP-3 alpha
57	MMP-2	4313	Gelatinase A
58	MMP-3	4314	Stromelysin-1
59	MMP-9	4318	CLG4B, Gelatinase B, GELB
60	MSP/MST1	4485	HGFL, MST1, SF2
61	MUC-1	4582	CD227, Episialin, H23AG, KL-6, Mucin-1, PEM, PEMT
62	Nectin-4	81607	LNIR, PRR4, PVRL4
63	Osteopontin (OPN)	6696	Eta-1, Spp1
64	p27/Kip1	1027	CDKN1B
65	p53	7157	BCC7, LFS1, TP53, TRP53
66	PDGF-AA	5154	
67	CD31/PECAM-1	5175	PECAM1
68	Progesterone R/NR3C3	5241	
69	Progranulin	2896	Acrogranin, GEP, GP88, GRN, PCDGF, PEPI, PGRN, Proepithelin
70	Prolactin	5617	PRL
71	Prostasin/Prss8	5652	
72	E-Selectin/CD62E	6401	ELAM1, LECAM2, SELE
73	Serpin B5/Maspin	5268	PI5
74	Serpin E1/PAI-1	5054	Nexin, PLANH1
75	Snail	6615	SLUGH2, SNAH, SNAI1
76	SPARC	6678	BM-40, Osteonectin
77	Survivin	332	API4, BIRC5
78	Tenascin C	3371	Cytotactin, HXB, Tenascin J1, TNC
79	Thrombospondin-1	7057	THBS1, TSP-1
80	Tie-2	7010	
81	u-Plasminogen Activator/Urokinase	5328	PLAU, uPA
82	VCAM-1/CD106	7412	
83	VEGF	7422	VAS, Vasculotropin, VEGFA, VPF
84	Vimentin	7431	Vimentin

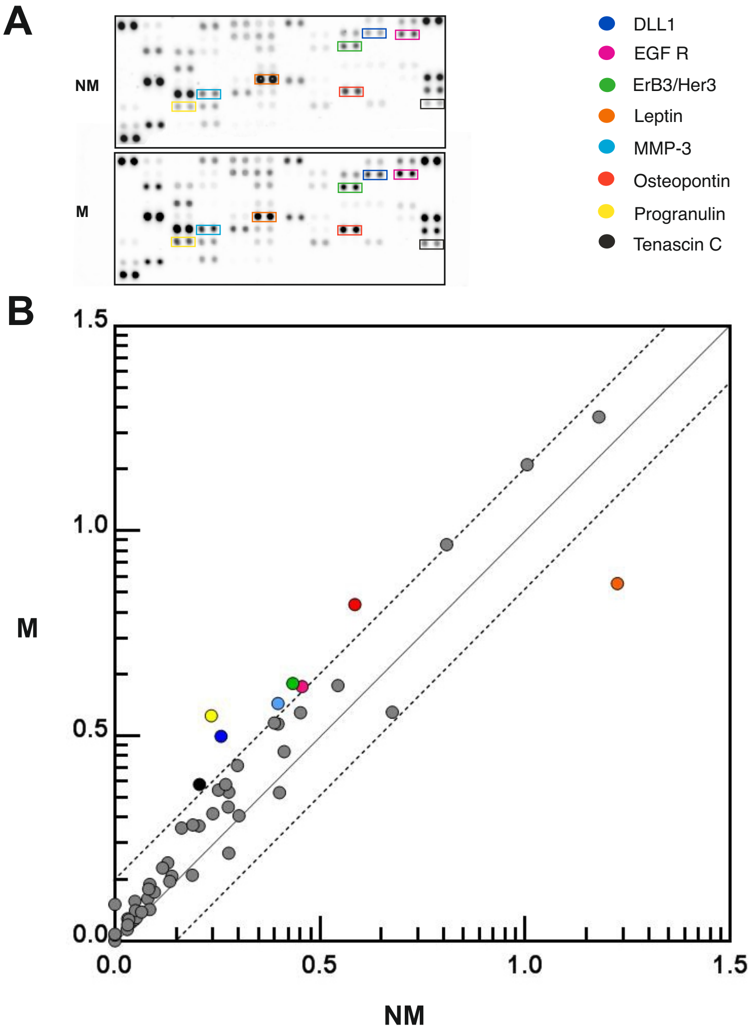

Supplementary figure 1. Representative proteins profile from pooled serum samples obtained at the time of primary uveal melanoma diagnosis. A) Dot blots of 84 cancer-related proteins from non-metastatic (NM) and metastatic (M) groups. B) Proteins were selected for further analysis if they 1) deviated between the pool of metastatic and non-metastatic patients, and 2) did not have a similar biological function as another protein with higher deviation between the groups Grey-colored dots represent proteins below the cut-off level.

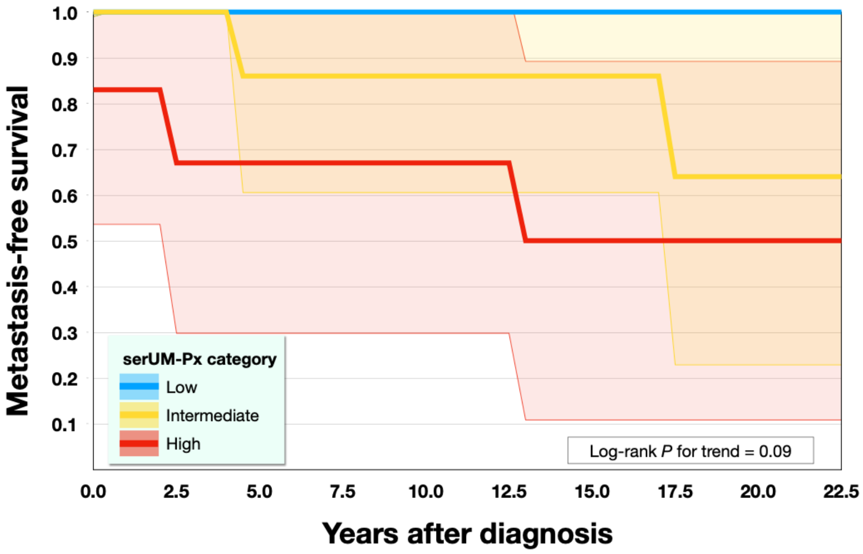

Number at risk						
Low	5	4	4	4	4	1
Intermediate	7	6	5	4	3	3
High	6	4	4	3	2	1

Supplementary figure 2. Kaplan-Meier metastasis-free survival by serUM-Px category in the training cohort (*n*=18).

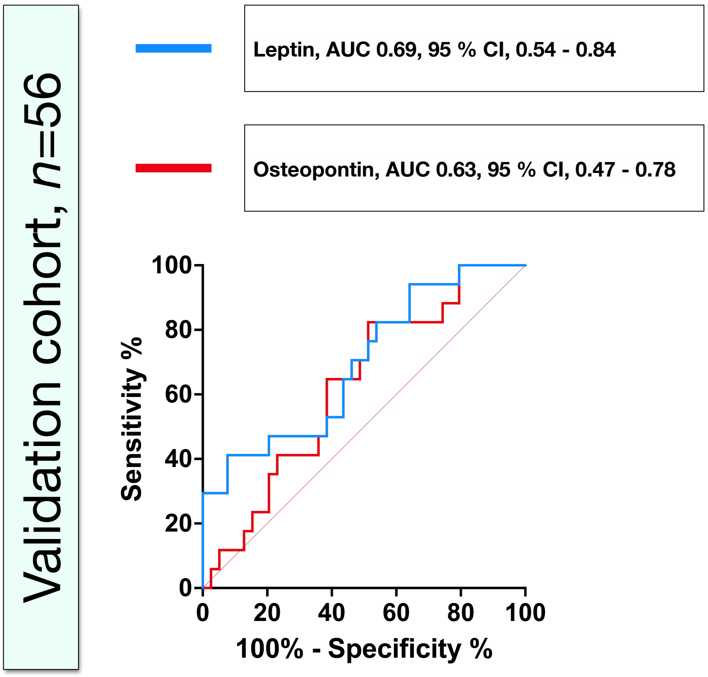

Supplementary figure 3. Receiver operating characteristics of leptin and osteopontin in the validation cohort. AUC, area under the curve. CI, confidence interval.

**Supplementary table 2.** serUM-Px metastatic risk category versus primary tumor BAP-1 expression			
**serUM-Px category**	**BAP-1 high, n**	**BAP-1 low, n**	***P****
**Low, n**	2	0	0.056
**Intermediate, n**	3	0	
**High, n**	3	4	

*Kruskal-Wallis test.

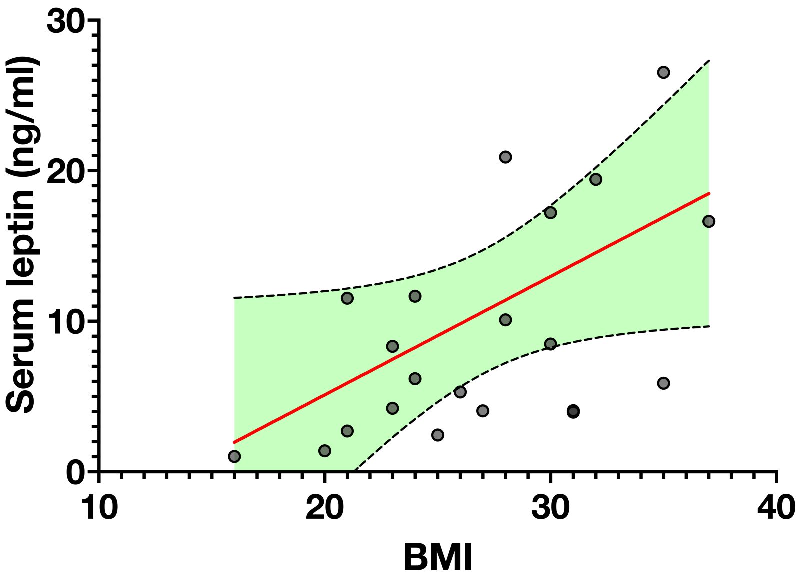

Supplementary figure 4. Linear regression of serum leptin levels versus patients' body mass index (BMI). Serum leptin levels had a positive correlation with BMI (standardized slope coefficient 0.79, *P*=0.041).

Supplementary table 3. Multivariate Cox regression, hazard for metastasis

**Multivariate**	**B**	**S.E.**	**Wald**	***P***	**Exp(B)**	**95 % CI lower**	**95 % CI upper**
Patient sex, male vs female	0.4	1.2	0.1	0.75	1.5	0.1	16.1
Serum leptin[a], ng/ml	0.1	0.1	0.4	0.51	1.1	0.9	1.2
BMI	-0.1	0.2	0.7	0.40	0.9	0.7	1.2

[a]Per increasing ng/ml. BMI, Body mass index.
